# Analysis of immunization time, amplitude, and adverse events of seven different vaccines against SARS-CoV-2 across four different countries

**DOI:** 10.3389/fimmu.2022.894277

**Published:** 2022-07-28

**Authors:** Maria Elena Romero-Ibarguengoitia, Arnulfo González-Cantú, Chiara Pozzi, Riccardo Levi, Maximiliano Mollura, Riccardo Sarti, Miguel Ángel Sanz-Sánchez, Diego Rivera-Salinas, Yodira Guadalupe Hernández-Ruíz, Ana Gabriela Armendariz-Vázquez, Gerardo Francisco Del Rio-Parra, Irene Antonieta Barco-Flores, Rosalinda González-Facio, Elena Azzolini, Riccardo Barbieri, Alessandro Rodrigo de Azevedo Dias, Milton Henriques Guimarães Júnior, Alessandra Bastos-Borges, Cecilia Acciardi, Graciela Paez-Bo, Mauro Martins Teixeira, Maria Rescigno

**Affiliations:** ^1^ Research Department, Hospital Clínica Nova de Monterrey, San Nicolás de los Garza, Nuevo Leon, Mexico; ^2^ Vicerrectoría de Ciencias de la Salud, Escuela de Medicina, Universidad de Monterrey, San Pedro Garza García, Mexico; ^3^ Instituti di Ricovero e Cura a Carattere Scientifico (IRCCS) Humanitas Research Hospital, Milan, Italy; ^4^ Department of Biomedical Sciences, Humanitas University, Milan, Italy; ^5^ Department of Electronic, Information and Bioengineering, Politecnico di Milano, Milan, Italy; ^6^ Health Department, Ternium Brazil, Rio de Janeiro, Brazil; ^7^ Research Department, Fundação São Francisco Xavier, Ipatinga, Brazil; ^8^ Health Secretary, Unidad Hospitalaria San José, Campana, Argentina; ^9^ Laboratory Department, Hospital Interzonal General de Agudos San Felipe, San Nicolás de los Arroyos, Argentina; ^10^ Biochemistry and Immunology Department, ICB, Universidade Federal de Minas Gerais, Belo Horizonte, Brazil

**Keywords:** COVID-19, SARS-CoV-2, immunization, vaccines, seroconversion

## Abstract

**Background:**

Scarce information exists in relation to the comparison of seroconversion and adverse events following immunization (AEFI) with different SARS-CoV-2 vaccines. Our aim was to correlate the magnitude of the antibody response to vaccination with previous clinical conditions and AEFI.

**Methods:**

A multicentric comparative study where SARS-CoV-2 spike 1-2 IgG antibodies IgG titers were measured at baseline, 21-28 days after the first and second dose (when applicable) of the following vaccines: BNT162b2 mRNA, mRNA-1273, Gam-COVID-Vac, Coronavac, ChAdOx1-S, Ad5-nCoV and Ad26.COV2. Mixed model and Poisson generalized linear models were performed.

**Results:**

We recruited 1867 individuals [52 (SD 16.8) years old, 52% men]. All vaccines enhanced anti-S1 and anti-S2 IgG antibodies over time (p<0.01). The highest increase after the first and second dose was observed in mRNA-1273 (p<0.001). There was an effect of previous SARS-CoV-2 infection; and an interaction of age with previous SARS-CoV-2 infection, Gam-COVID-Vac and ChAdOx1-S (p<0.01). There was a negative correlation of Severe or Systemic AEFI (AEs) of naïve SARS-CoV-2 subjects with age and sex (p<0.001); a positive interaction between the delta of antibodies with Gam-COVID-Vac (p=0.002). Coronavac, Gam-COVID-Vac and ChAdOx1-S had less AEs compared to BNT162b (p<0.01). mRNA-1273 had the highest number of AEFIs. The delta of the antibodies showed an association with AEFIs in previously infected individuals (p<0.001).

**Conclusions:**

The magnitude of seroconversion is predicted by age, vaccine type and SARS-CoV-2 exposure. AEs are correlated with age, sex, and vaccine type. The delta of the antibody response only correlates with AEs in patients previously exposed to SARS-CoV-2.

**Registration number:**

ClinicalTrials.gov, identifier NCT05228912.

## Introduction

Covid-19 is a pneumonia-like disease caused by a coronavirus, named SARS-CoV-2. It is highly contagious, and the World Health Organization declared it in 2020 as a pandemic (1). SARS-CoV-2 caused COVID-19 that has a wide range of clinical presentations, from asymptomatic disease to severe acute respiratory distress syndrome and death (2). SARS-CoV-2 utilizes its surface spike glycoprotein to enter host cells. Each unit of the spike trimer contains an S1 and S2 subunit, with the N-terminal S1 subunit binding to the cellular angiotensin-converting enzyme 2 (ACE2) receptor through an internal receptor-binding domain (RBD) (3).

To prevent SARS-CoV-2 infection, 113 vaccines are being tested in human clinical trials, and 44 have reached the final step of testing (4). Based on the mechanism of action, vaccines can be clustered in four groups: 1) mRNA vaccines, for example, BNT162b2 mRNA and mRNA-1273. They use genetically engineered modified RNA to produce the spike protein that safely prompts an immune response safely. 2) Viral vector (adenovirus) vaccines, for example, ChAdOx1-S, Ad26.COV2, Ad5-nCoV, and Gam-COVID-Vac. They use a virus that has been genetically engineered so that it cannot cause disease but produces coronavirus proteins to safely generate an immune response. 3) Inactivated virus vaccines, for example, Coronavac, use a form of the virus that has been inactivated or weakened, so it does not cause disease but still generates an immune response. 4) Protein subunits vaccines, for example, NVX-CoV2373. It is a protein-based vaccine that uses harmless fragments of proteins or protein shells that mimic the SARS-CoV-2 to safely generate an immune response (5).

Currently BNT162b2 mRNA, mRNA-1273, ChAdOx1-S, Ad26.COV2, Ad5-nCoV, Gam-COVID-Vac, Coronavac, have been administered to people across 587 countries (4). However, no studies have compared using the same assay and time frame the effectiveness of seroconversion and incidence of adverse events in response to vaccines in different countries in the same study.

This study aimed to correlate the magnitude of the antibody response after the first and second dose (if applicable) between BNT162b2 mRNA, mRNA-1273, ChAdOx1-S, Ad26.COV2, Ad5-nCoV, Gam-COVID-Vac, and Coronavac by measuring SARS-CoV-2 spike 1-2 IgG antibodies, using the same standardized assay, with previous clinical conditions and assessing systemic and severe adverse events (AEs).

## Materials and methods

This was a multicentric observational study of volunteers who received an approved complete scheme of BNT162b2 mRNA, mRNA-1273, ChAdOx1-S, Ad26.COV2, Ad5-nCoV, Gam-COVID-Vac, or Coronavac COVID-19 vaccine during 2021 in five hospital centers (Hospital Clinica Nova, Humanitas Clinical and Research Center, Fundacion San Francisco Xavier, Ternium Health Center in Rio, Hospital Municipal San Jose, Hospital Interzonal de Agudos San Felipe) from four different countries: Mexico, Italy, Brazil and Argentina. This study followed STROBE guidelines (6). The study was approved by each local Institutional Review Board and conducted per the Code of Ethics of the World Medical Association (Declaration of Helsinki) for experiments that involve humans.

The inclusion criteria were volunteers of both genders, any age, who consented to participate, planned to conclude the immunization regimen of any vaccine, and agreed to be followed up for the duration of the study. The following vaccines were available: BNT162b2 mRNA, mRNA-1273, ChAdOx1-S, Ad26.COV2, Ad5-nCoV, Gam-COVID-Vac, or Coronavac. The exclusion criteria were to have received any SARS-CoV-2 vaccine prior to study entry.

Each country’s Health System defined the available vaccines, the schedule, and dose assignation. On the vaccination day, the research team invited any subject who planned to receive any vaccine scheme, explained the project and asked to sign the informed consent. Inclusion-exclusion criteria were applied, and a plasma sample was collected. The baseline sample was taken before receiving the first dose of any SARS-CoV-2 vaccine (T0). The second (T1) and third samples (T2) were taken after 21 days (+/- 7 days) of the first and second dose, respectively.

At each visit, participants had to answer a questionnaire. The basal questionnaire aimed at obtaining patients’ medical history and previous SARS-CoV-2 infections. The questionnaires applied after the first and second dose of vaccines aimed at recognizing adverse events following immunization (AEFI) (7) and identifying a potential SARS-CoV-2 infection after receiving any vaccine dose. SARS-CoV-2 infection was also monitored by the epidemiology team through PCR testing, which informed the research team of any new infection.

### Primary outcome, IgG determination

Our primary outcome was to correlate the magnitude of the antibody response to vaccination with previous clinical conditions and AEs . To determine the amount of specific anti-S1 and anti-S2 IgG antibodies against SARS-CoV-2 in plasma samples, the laboratory personnel used a chemiluminescence immunoassay (CLIA) developed by DiaSorin, which had a sensitivity of 97.4% (95% CI, 86.8-99.5) and a specificity of 98.5% (95% CI, 97.5-99.2). The results were reported as follows: <12.0 AU/ml was considered negative, 12.0 to 15.0 AU/ml was indeterminate, and > 15 AU/ml was positive. This kit is comparable with other commercial kits and has been used elsewhere (8–11).

The variables we analyzed were age, sex, personal medical history (for example, the presence of type 2 diabetes, hypertension, obstructive pulmonary disease, any heart condition, obesity, cancer, liver steatosis, any autoimmune disease), and confirmed SARS-CoV-2 (through nasal swab or serologic tests such as IgG determination). The following AEs were considered of particular interest: fever (>37.5°C), adenopathy, diffuse rash, edema, facial paralysis, orthostatic hypotension, headache, arthralgia, myalgia, nausea, vomit, and diarrhea. Anti-S1 and anti-S2 IgG antibodies against SARS-CoV-2 were measured at baseline, 21-28 days post-first dose (S1), and 21-28 days post-second dose if applicable.

### Statistical methods

The researchers reviewed the quality control and the anonymization of the database. Normality assumption was evaluated with the Shapiro-Wilk test or Kolmogorov. Descriptive statistics such as mean, standard deviation, median, interquartile range, frequencies, and percentages were computed. Kruskal-Wallis test with Dunn’s multiple comparisons test was performed for IgG comparisons. We performed a mixed model where the dependent variable was the delta of the antibodies. The personal variability and time were constructed as a random effect, whereas each type of vaccine, age, history of SARS-CoV-2, and the interaction of each vaccine with age were fixed effects. BNT162b mRNA was the reference vaccine group. For the analysis of AEs, the study population was divided into two separate cohorts by stratifying according to the history of SARS-CoV-2 infection (1050 Naïve SARS-CoV-2 subjects and 471 SARS-CoV-2 history subjects). Poisson generalized linear models (PGLM) were performed on both cohorts with the counts of AEs events after the second dose as the outcome variables. Included regressors were age, sex, body mass index, 1,000 AU/mL variation of IgG levels after the second dose compared to the baseline IgG levels, type of vaccine and the interactions between vaccines and IgG level variation. BNT162b mRNA was the reference vaccine group Missing completely at random values were analyzed through complete case analysis since missing antibody levels were less than 5% and we considered it incorrect to impute any AEFI. A sample size of 1870 patients was calculated, according to the primary aim, by using a mixed model formula with an alpha of 0.05, power of 90%, the effect size of 0.15, and k=7. The statistical programs used were R v.4.0.3 and Python v. 3.8.3. The analyses were two-tailed. A p-value less than 0.05 was statistically significant.

## Results

A total of 1867 patients were recruited from all countries: 1352 from Mexico, 42 from Italy, 260 from Brazil, and 213 from Argentina. The most frequent vaccine used was ChAdOx1-S in 666 subjects, Coronavac in 582, BNT162b2 mRNA in 289, Gam-COVID-Vac in 213, mRNA-1273 in 65, Ad26.COV2 in 31, and Ad5-nCoV in 19. The mean (SD) age was 52 (16.8) years, being statistically different across vaccine groups (p<0.05) as some vaccines were proposed to a particular age group. Fifty-two % of subjects were men, 559 (30%) had obesity, and 501 (26.8%) had hypertension. **Table 1** shows the medical history of patients divided by vaccine type.

**Table 1:** General characteristics and medical history.

	BNT162b2 mRNA (%)	mRNA-1273 (%)	Gam-COVID-Vac (%)	Coronavac (%)	ChAdOx1-S (%)	Ad5-nCoV (%)	Ad26.COV2 (%)	p-value
Total	289	65	200	582	666	19	31	
Obesity	54 (18.6)	11 (16.9)	44 (22)	188 (32.3)	248 (37.2)	8 (42.1)	6 (19.3)	<0.001
Smoker	37 (12.8)	10 (15.3)	27 (13.5)	60 (10.3)	38 (5.7)	2 (10.5)	2 (6.4)	0.001
Hypertension	36 (12.4)	2 (3)	77 (38.5)	116 (19.9)	265 (39.7)	3 (15.7)	2 (6.4)	<0.001
Dyslipidemia	27 (9.3)	3 (4.6)	55 (27.5)	76 (13.5)	150 (22.5)	1 (5.2)	1 (3.2)	<0.001
Type2 Diabetes	13 (4.4)	1 (1.5)	32 (16)	78 (13.4)	164 (24.6)	0	0	<0.001
Asthma	9 (3.1)	3 (4.6)	6 (3)	13 (2.3)	17 (2.5)	0	0	0.79
Other autoimmune disease such as thyroiditis or psoriasis	9 (3.1)	0	4 (2)	29 (4.9)	60 (9)	1 (5.2)	1 (3.2)	<0.001
Rheumatoid arthritis	7 (2.4)	1 (1.5)	16 (8)	11 (1.8)	56 (8.4)	0	1 (3.2)	<0.001
Surgery in the last year	6 (2)	0	6 (3)	26 (4.4)	45 (6.7)	3 (15)	1 (3.2)	0.002
Previous Cancer	5 (1.7)	1 (1.5)	1 (0.5)	5 (0.8)	29 (4.3)	0	0	0.001
NAFLD	4 (1.3)	0	7 (3.5)	27 (4.6)	21 (3.1)	2 (10.5)	0	0.042
Immunosuppressive treatment	4 (1.3)	0	3 (1.5)	6 (1)	11 (1.6)	0	0	0.86
Other liver disease	3 (1)	0	10 (5)	10 (1.7)	10 (1.5)	0	0	0.02
End stage renal disease	2 (0.7)	0	2 (1)	3 (0.5)	11 (1.6)	0	0	0.44
Pregnancy	2 (0.7)	1 (1.5)	0	3 (0.5)	0	1 (5.2)	0	0.005
Cirrhosis	2 (0.7)	0	0	2 (0.3)	4 (0.6)	0	0	0.88
Pulmonar Obstructive Chronic Disease	1 (0.3)	0	3 (1.5)	4 (0.6)	8 (1.2)	0	0	0.68
Coronary heart disease	1 (0.3)	0	15 (7.5)	3 (0.5)	23 (3.4)	0	0	<0.001
Gout	1 (0.3)	0	10 (5)	19 (3.2)	27 (4.05)	0	0	0.02
Active Cancer	0	0	1 (0.5)	1 (0.17)	10 (1.5)	0	0	0.054
Atrial Fibrilation	0	0	13 (6.5)	6 (1)	36 (5.4)	0	0	0.001
Congestive heart failure	0	0	18 (9)	1 (0.2)	12 (1.8)	0	0	0.001
Stroke	0	0	2 (1)	1 (0.2)	13 (1.9)	0	0	0.01
Trasplant	0	0	0	3 (0.5)	5 (0.7)	0	0	0.63
Pregnancy	2	1	0	3 (0.5)	0	1 (5.2)	0	0.05

**Table 1** shows the medical history and comparison between vaccine groups, p<0.05 was considered statistically significant.

### History of SARS-CoV-2

Positive history of SARS-CoV-2 infection was considered if the volunteer had a confirmatory swab or IgG positive serological test at baseline. Additionally, we considered a positive SARS-CoV-2 history if specific anti-S1 and anti-S2 IgG antibodies against SARS-CoV-2 in plasma samples were > 15 AU/ml at baseline. We had a total of 627 positive cases before vaccination, of which 165 (24.8%) received ChAdOx1-S, 246 (42.2%) Coronavac, 107 (37.1%) BNT162b2 mRNA, 55 (25.8%) Gam-COVID-Vac, 31 (47.6%) mRNA-1273, 18 (58%) Ad26.COV2, and 5 (26%) Ad5-nCoV.

### Antibody titers

All vaccines showed significant anti-S1 and anti-S2 IgG antibody changes with significant differences across vaccines and depending on SARS-CoV-2 history. In naïve patients, the highest increase [Median (IQR) AU/ml] after the first dose was observed in mRNA-1273 [175.5 (76.7)], then BNT162b mRNA [78.1 (55)], and Ad5-nCoV [43.4(55.9)]. In subjects previously exposed to SARS-CoV-2, the highest increase after the first dose was observed in mRNA-1273 [5920(4705)], then BNT162b mRNA [2500(3080)], and ChAdOx1-S [1025(2489)].

**Figure 1:**
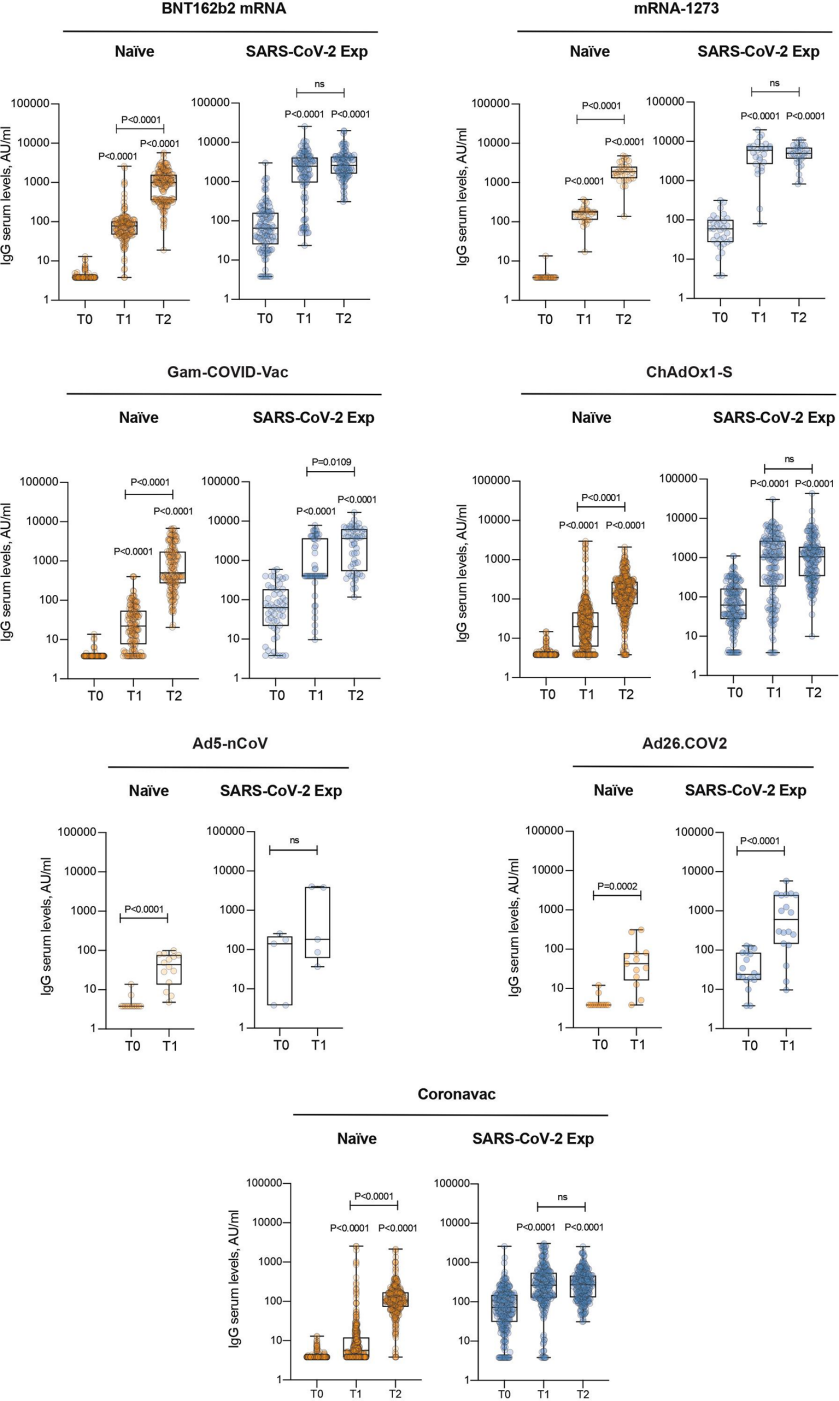
IgG antibody levels by vaccine. IgG antibody response was measured in serum of naïve and SARS-CoV-2 previously exposed (SARS-CoV-2 Exp) subjects at different time points (T0, T1 and T2) and vaccinated with different vaccine types. Samples ≥ 15 AU/mL were considered positive. Log scale on y axis. The box plots show the interquartile range, the horizontal lines show the median values, and the whiskers indicate the minimum-to-maximum range. Each dot corresponds to an individual subject. P-values were determined using 2-tailed Kruskal-Wallis test with Dunn’s multiple comparisons test. P-values refer to baseline (T0) when there are no connecting lines. ns, not significant.

In naïve patients, the highest increase [Median (IQR) AU/ml] after the second dose (T2) was observed in mRNA-1273 [1875 (1190)], then BNT162b mRNA [998 (1241)], and Gam-COVID-Vac [501 (55.9)], whereas in SARS-CoV-2 previously exposed subjects the highest increase after the second dose was observed in mRNA-1273 [4950(3060)], then Gam-COVID-Vac [3620 (5356)], and BNT162b mRNA [2630 (2672.5)]. **Figure 1**, **Supplementary Figure S1** show the change in antibody levels through time, classified by vaccine type. **Table 2** shows the antibody levels classified by vaccine type.

**Table 2:** Antibody differences between vaccine types.

	SARS CoV-2 Naïve							
Vaccine Median (IQR)	BNT162b2 mRNA (n=289)	mRNA-1273 (n=65)	Gam-COVID-Vac (n=213)	Coronavac (n=582)	ChAdOx1-S (n=666)	Ad5-nCoV Cansino (n=19)	Ad26.COV2(n=31)	p-value
Basal	3.8 (0)	3.8 (26.6)	3.8 (0)	3.8(0.9)	3.8 (0)	3.8 (0)	3.8 (13.5)	p<0.005
After First Dose	78.1 (55.0)	175.5 (76.7)	22.1 (44.5)	5.59 (8.0)	19.9 (39.6)	43.45 (55.9)	42.6 (57.5)	p<0.001
After Second Dose	998 (1241.0)	1875 (1190.0)	501 (1453.7)	109 (97.6)	140.5 (199.5)	NA	NA	p<0.001
	**SARS CoV-2 Positive**							
Basal	66.9 (130.8)	59.3 (67.9)	59.8 (157.3)	72.4 (119.0)	61.9 (134.8)	141.0 (175.2)	24 (66.7)	p<0.001
After First dose	2500.0 (3080.0)	5920.0 (4705.0)	400.0 (3252.0)	264.5 (422.7)	1025.0 (2489.0)	182.0 (3734.6)	606.5 (23.69.0)	p<0.001
After Second Dose	2630.0 (2672.5)	4950.0 (3060)	3620 (5356)	279(337)	1020 (1538)	NA	NA	p<0.001

BNT162b2 mRNA, mRNA-1273 and Gam-COVID-Vac showed higher antibody levels after first and second dose.

*Kruskal-Wallis test was performed for comparison, p<0.05 was considered statistically significant. NA, does not apply.

**Figure 2:**
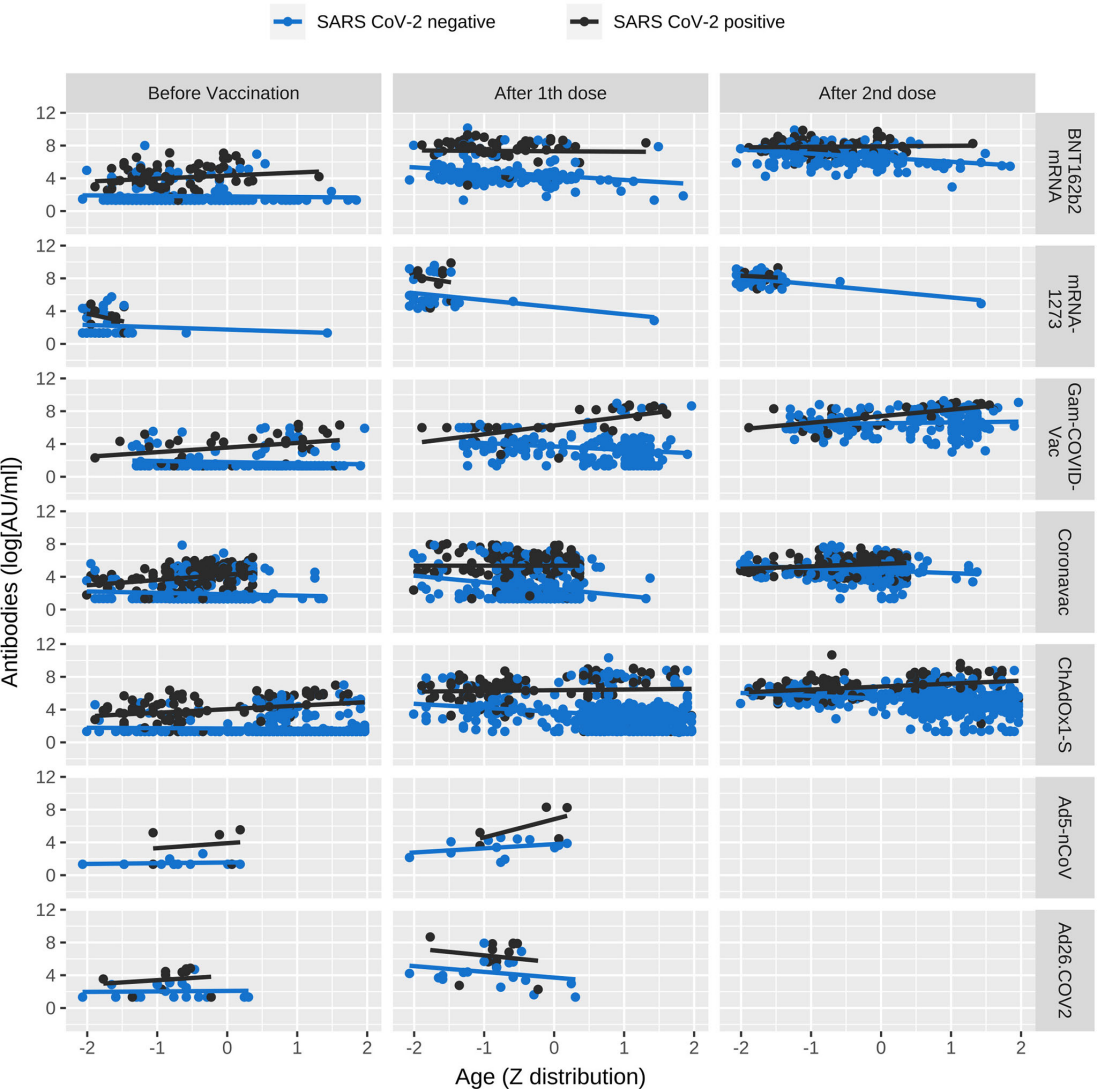
Antibody titers classified by age type of vaccine and Time. Figure shows change in antibody concentration through age, divided SARS-CoV-2 history, and vaccine type. Antibodies are expressed in logarithm and age has a Z distribution.

The mixed model that showed a positive effect of SARS-CoV-2 previous infection (B=767.468, p<0.001), a positive interaction of age with previous SARS-CoV-2 infection (B=13.03, p=0.003), and an interaction of Gam-COVID-Vac and ChAdOx1-S with age (B=46.5, p<0.001; B=20.5; p=0.006). With respect to BNT162b mRNA, mRNA-1273 showed a higher change in antibody titers, while all the other vaccines had less change (p<0.01). We computed sex in the model, but there was no significant correlation, so we eliminated it from the final model. These results are shown in **Table 3** and **Figure 2**.

**Table 3:** Mixed model of antibody changes.

	Estimate	Std. Error	95% CI	t value	p-value
(Intercept)	1909.02	345.73	1271.81 – 2546.23	5.52	<0.001
Age	-19.40	6.79	-32.6 7– -6.11	-2.855	0.004
SARS CoV2 previous infection	767.46	231.84	314.45 – 1220.31	3.31	0.001
mRNA-1273	1855.79	501.63	876.08 – 2836.04	3.69	<0.001
Gam-COVID-Vac	-2768.04	482.86	-3711.06 – -1824.4	-5.73	<0.001
Coronavac	-2184.03	430.93	-3025.82– -1342.09	-5.06	<0.001
ChAdOx1-S	-1808.26	361.77	-2514.69 – -1101.2	-4.99	<0.001
Ad5-nCoV	-2710.10	1542.89	-5727.76 – 304.30	-1.75	0.079
Ad26.COV2	-791.46	1291.27	-3317.21 – 1731.09	-0.613	0.54
Age*mRNA-1273	-20.82	15.95	-52 – 10.34	-1.3	0.192
Age*Gam-COVID-Vac	46.57	9.15	28.69 – 64.44	5.09	<0.001
Age*Coronavac	16.78	9.44	-1.67 – 35.22	1.77	0.076
Age*ChAdOx1-S	20.51	7.46	5.92 – 35.08	2.749	0.006
Age*Ad5-nCoV	44.44	35.86	-25.68 – 114.54	1.23	0.215
Age*Ad26.COV2	-4.24	32.52	-67.82 – 59.33	-0.13	0.896
Age*COVID-19 infection	13.03	4.44	4.35 – 21.71	2.93	0.003

Mixed model. The dependent variable is the delta of antibodies. Time and personal variability are random effects. Reference group BNT162b2 mRNA. Previous exposure to SARS-CoV-2 were related to a higher antibody change. Older subjects that received Gam-COVID-Vac or ChAdOx1-S had higher antibody change.* means "interaction", i.e. the product of the variables.

### Adverse events following immunization

**Figure 3:**
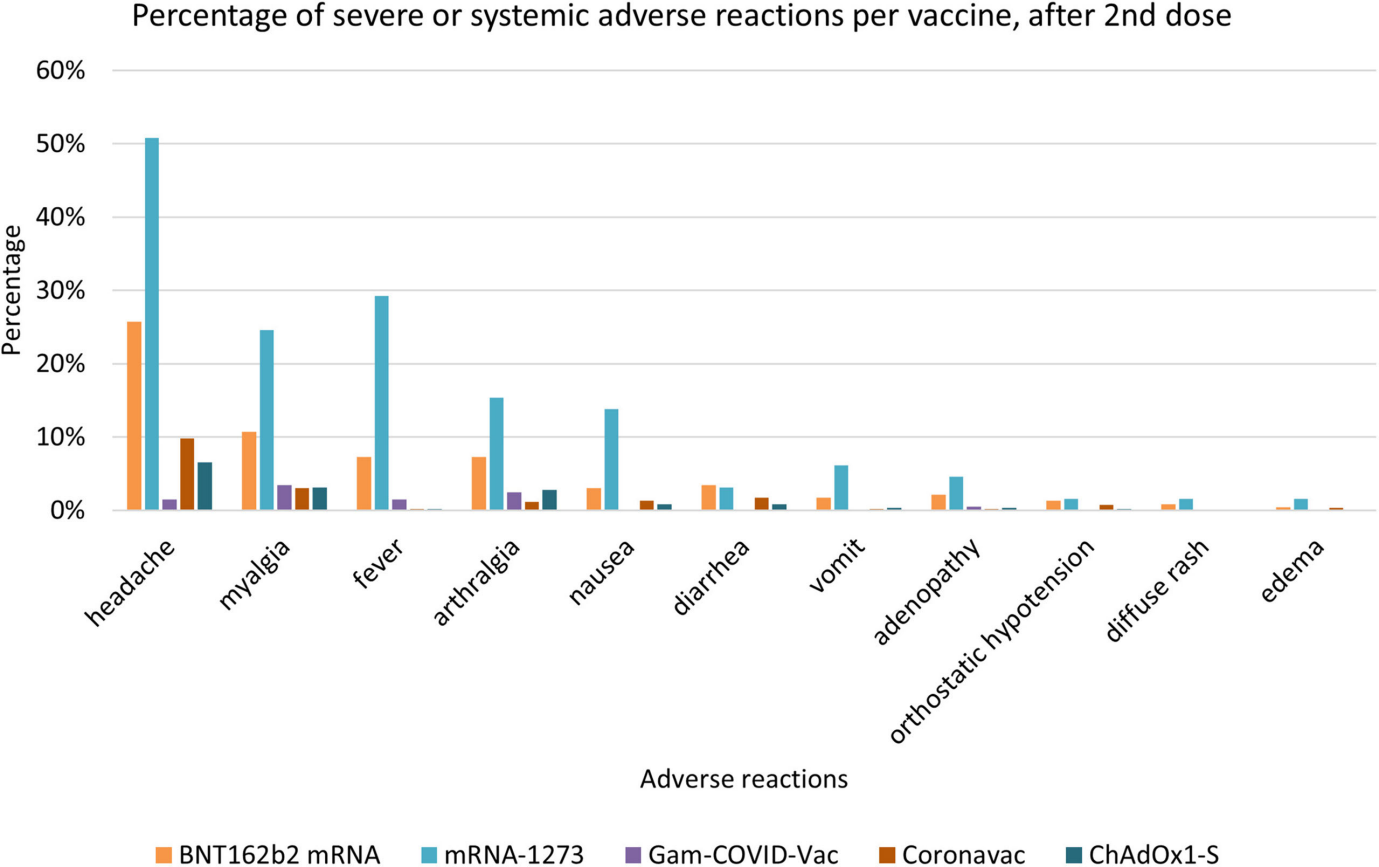
Percentage of systemic or severe adverse events after the second dose, stratified by vaccine. The graph shows the frequency of appearance of each systemic or severe adverse event across the considered vaccine groups.

At least one AEFI was observed after the first dose in 71% of respondents receiving BNT162b2 mRNA, in 93% with mRNA-1273, 38% with Gam-COVID-Vac, 42% with Coronavac, 51% with ChAdOx1-S, 74% with Ad5-nCoV, and 81% with Ad26.COV2. After the second dose, 65%, 88%, 23%, 33%, and 23% of the respondents experienced at least one AEFI after receiving BNT162b2 mRNA, mRNA-1273, Gam-COVID-Vac, Coronavac, and ChAdOx1-S, respectively. For each vaccine, the majority of the adverse events occurred during the first 24 hrs after injection, either after the first or the second dose. Patients receiving BNT162b2 mRNA, mRNA-1273, Gam-COVID-Vac, Coronavac, ChAdOx1-S, Ad5-nCoV, and Ad26.COV2 subjectively qualified the AEFI after the first dose as “very mild” or “mild” in 85%, 80%, 95%, 84%, 67%, 93%, and 57% of cases, respectively. The AEFI after the second dose were qualified as “very mild” or “mild” by 82%, 49%, 98%, 89%, and 76% of patients receiving BNT162b2 mRNA, mRNA-1273, Gam-COVID-Vac, Coronavac, and ChAdOx1-S respectively. Finally, 49% of patients receiving mRNA-1273 qualified for their adverse events after the second dose as “moderate”. **Figure 3** shows the percentages of AEs per vaccine.

AEs were evaluated by using PGLM. The model for naïve SARS-CoV-2 subjects showed a negative association of the count of adverse events with age (B=-0.0327, p<0.001) and sex (B=-0.8145, p<0.001). A negative effect with respect to BNT162b mRNA was observed for Gam-COVID-Vac (B=-1.8582, p<0.001), Coronavac (B=-0.5736, p=0.047) and ChAdOx1-S (B=-0.7871, p=0.006). Finally, a positive interaction was found between the delta of antibodies due to vaccination and Gam-COVID-Vac (B=0.5604, p=0.002).

**Figure 4:**
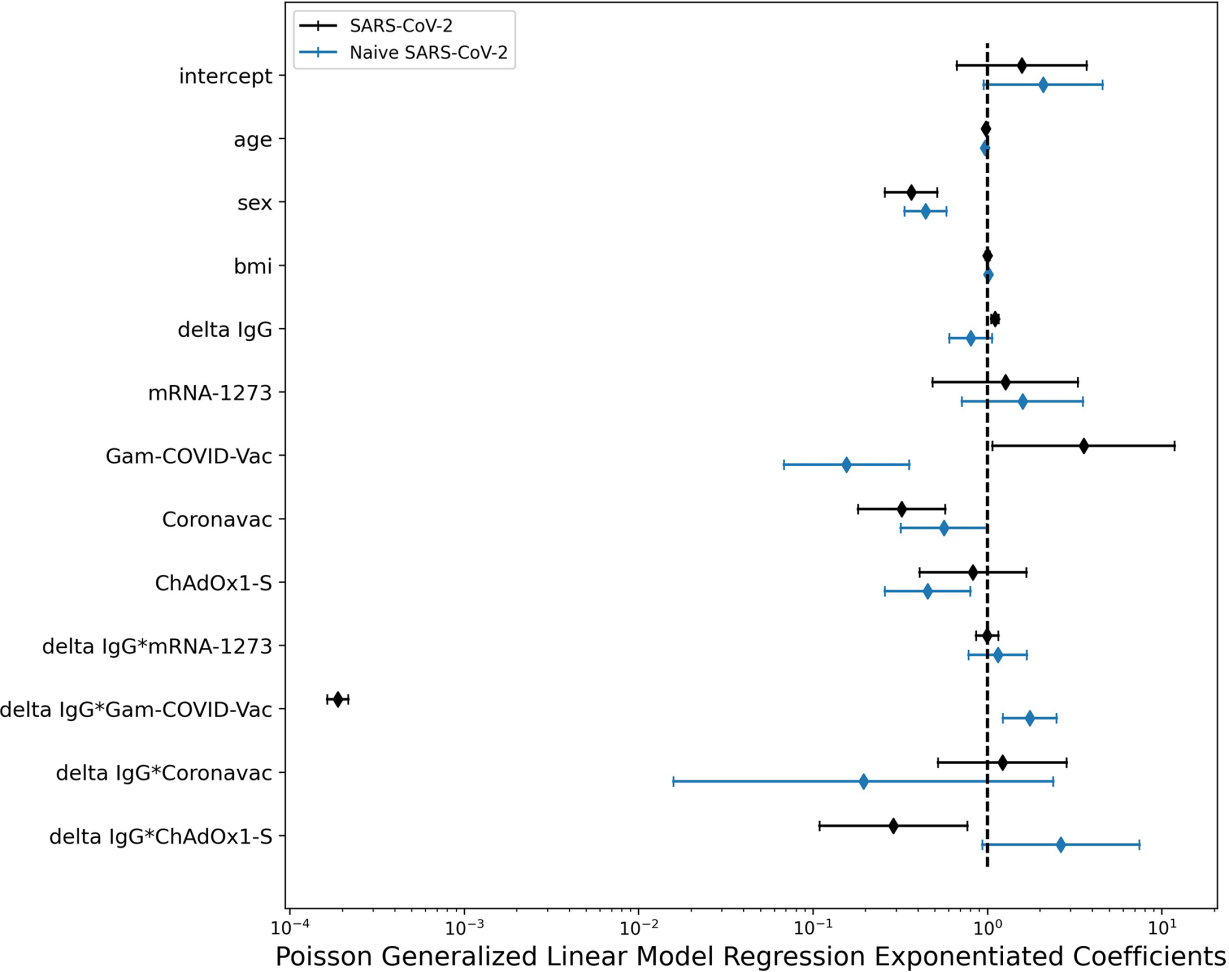
Forest plot for the GLM Poisson model coefficients stratified by SARS-CoV-2 history. Women and young individuals developed more severe or systemic AEFI (AEs). There was no effect of Body Mass Index. In the naïve SARS-CoV-2 cohort, the vaccines Gam-COVID-Vac, Coronavac and ChAdOx1-S were related to fewer AEs after the second dose than BNT162b mRNA. However, for naïve SARS-CoV-2 patients who received Gam-COVID-Vac, higher antibody levels after the second dose were related to a greater number of AEs. In people previously exposed to SARS-CoV-2, those receiving Gam-COVID-Vac showed a greater number of AEs compared to BNT162b mRNA, while subjects receiving Coronavac showed significantly fewer events when compared to BNT162b mRNA.

The model for subjects previously exposed to SARS-CoV-2 showed a negative association of the count of adverse events with age (B=-0.0190, p=0.013) and sex (B=-1.0042, p<0.001). The delta of the antibodies showed a positive association with the count of adverse events (B=0.1018, p<0.001). A positive effect with respect to BNT162b mRNA was observed for Gam-COVID-Vac (B=1.2737, p=0.038), whereas a negative effect was observed for Coronavac (B=-1.1304, p<0.001). Negative interactions were also found between the delta of antibodies due to vaccination and the vaccines Gam-COVID-Vac (B=-8.5758, p<0.001) and ChAdOx1-S (B=-1.2386, p=0.013). See **Tables 4**, **5** and **Figure 4** for details.

**Table 4:** Number of systemic or severe adverse events following vaccination in SARS-CoV-2 Naïve subjects.

	*β*	e^*β*^	95% CI	p-value
Intercept	0.73	2.08	0.95-4.57	0.066
Age	-0.03	0.96	0.95-0.97	<0.001
Sex (M)	-0.81	0.44	0.33-0.58	<0.001
Body Mass Index	0.015	1.01	0.99-1.03	0.175
Delta IgG	-0.21	0.80	0.60-1.06	0.128
mRNA-1273	0.46	1.59	0.71-3.54	0.255
Gam-COVID-Vac	-1.85	0.15	0.06-0.35	<0.001
Coronavac	-0.57	0.56	0.31-0.99	0.047
ChAdOx1-S	-0.78	0.45	0.25-0.79	0.006
Delta IgG*mRNA-1273	0.13	1.14	0.78-1.68	0.479
Delta IgG*Gam-COVID-Vac	0.56	1.75	1.22-2.49	0.002
Delta IgG*Coronavac	-1.63	0.19	0.01-2.39	0.201
Delta IgG*ChAdOx1-S	0.97	2.64	0.93-7.44	0.066

Poisson Generalized Linear Model regression. The reference group is BNT162b2 mRNA. Women and young individuals developed more severe or systemic AEFI (AEs). There was no effect of Body Mass Index. In the naïve SARS-CoV-2 cohort, the vaccines Gam-COVID-Vac, Coronavac and ChAdOx1-S were related to fewer AEs after the second dose than BNT162b mRNA. However, for naïve SARS-CoV-2 patients who received Gam-COVID-Vac, higher antibody levels after the second dose were related to a greater number of AEs.* means "interaction", i.e. the product of the variables.

**Table 5:** Number of systemic or severe adverse events following vaccination in SARS-CoV-2 previously exposed subjects. .

	*β*	e^*β*^	95% CI	p-value
Intercept	0.45	1.57	0.66-3.71	0.297
Age	-0.01	0.98	0.96-0.99	0.013
Sex (M)	-1.00	0.36	0.25-0.51	<0.001
Body Mass Index	0.004	1.00	0.97-1.03	0.776
Delta IgG	0.10	1.10	1.05-1.16	<0.001
mRNA-1273	0.23	1.26	0.48-3.31	0.628
Gam-COVID-Vac	1.27	3.57	1.07-11.89	0.038
Coronavac	-1.13	0.32	0.18-0.57	<0.001
ChAdOx1-S	-0.18	0.82	0.40-1.67	0.597
Delta IgG*mRNA-1273	-0.001	0.99	0.86-1.15	0.982
Delta IgG*Gam-COVID-Vac	-8.57	0.0001	0.00016-0.00022	<0.001
Delta IgG*Coronavac	0.19	1.22	0.52-2.84	0.644
Delta IgG*ChAdOx1-S	-1.23	0.28	0.10-0.76	0.013

Poisson Generalized Linear Model regression. Reference group is BNT162b2 mRNA. Women and young individuals developed more severe or systemic AEFI (AEs). There was no effect of Body Mass Index. In subjects previously exposed to SARS-CoV-2, those receiving Gam-COVID-Vac showed a greater number of AEs compared to BNT162b mRNA, while subjects receiving Coronavac showed significantly fewer events when compared to BNT162b mRNA.* means "interaction", i.e. the product of the variables.

### SARS-CoV-2 infection

Subjects were followed up to 28 days after the second dose administration. Between the first and second dose, one (0.34%) patient with BNT162b2 mRNA, 0 with mRNA-1273, 1 (0.46%) with Gam-COVID-Vac, 3 (0.51%) with Coronavac, 4 (0.6%) with ChAdOx1-S, 2 (10%) with Ad5-nCoV and 1 (3%) with Ad26.COV2 got the SARS-CoV-2 infection. After the second dose, 4 (1.3%) with BNT162b2 mRNA, 1 (1.5%) with mRNA-1273, 0 with Gam-COVID-Vac, 18 (3%) with Coronavac, and 11 (1.6%) with ChAdOx1-S became infected with SARS-CoV-2 (p<0.01).

## Discussion

This multicenter study compared the 21-28 day seroconversion, the AEFI after first and second doses, and the associated predictors of AEs of seven of the most common vaccines used worldwide: BNT162b2 mRNA, mRNA-1273, Gam-COVID-Vac, Coronavac, ChAdOx1-S, Ad5-nCoV, and Ad26.COV2 (4).

All vaccines showed immunogenicity and seroconversion after the complete vaccination scheme. The greatest change in antibody levels occurred with mRNA-1273, followed by BNT162b2 mRNA and Gam-COVID-Vac. Previous studies have shown a greater change in antibody titers for mRNA vector vaccines. This type of vaccines delivers the genetic information for the antigen, and vaccinated individuals synthesize antigens in the host cells (12–14).

Our study found that the previous history of SARS-CoV-2 was related to an increase in change in antibody levels as confirmed elsewhere (12). SARS-CoV-2 infection induces a robust humoral and cellular immune response. IgM, IgA, and IgG can be detected in the blood 5–15 days following symptom onset or a positive reverse transcriptase polymerase chain reaction (RT-PCR) test. Both binding and neutralizing antibody titers rise faster and reach a higher peak in patients with more severe COVID-19. SARS-CoV-2 vaccines result in early production of serum IgA, IgM, and IgG antibodies, and also induce long-lasting memory B- and T-cell responses (15). It seems that the presence of previous SARS-Cov-2 synergizes with all the vaccines tested in this study and offers a boost in antibody levels. This is a logical finding as the infection would be the first contact with the antigen and vaccination the second encounter with relevant antigens.

Previous studies found that persons aged 65-80 years and above have significantly lower peak of anti-S and neutralizing antibody titers following vaccination than those less than 65 years (16–18). We confirmed this result in our mixed model for most of the vaccines except for patients who had previous SARS-CoV-2 infection and received Gam-COVID or ChAdOx1-S, where we found higher antibody levels. There is limited literature to which to compare this finding; however, the study of Logunov et al. that tested the efficacy of Gam-COVID Vac reported a higher increase of anti-SARS-CoV-2 IgG levels in the group of 18-30 years and no particular change in other age groups (19); but they excluded patients with previous SARS-CoV-2 infection, so these results are not comparable. Whatever the explanation for this finding, it is interesting to note that older individual may benefit from vaccinating with adenoviral vector vaccines, such as Gam-COVID or ChAdOx1-S. However, in the study conducted by Ramasamy et al., similar antibody titers were seen 28 days after the boost vaccination of ChAdOx1 across all groups, regardless of age or vaccine dose (20). Therefore, it is difficult to take firm conclusions regarding the preferential use of this vaccine in older age groups.

Increasing age has been associated with decreased likelihood of seroconversion but higher peak antibody titers among those who do seroconvert after SARS-CoV-2 infection (21). Also, both binding and neutralizing antibody titers rise faster and reach a higher peak in patients with more severe COVID-19 (21–23). It is possible that older patients in our group of the study had a more severe disease during SARS-CoV-2 infection and that they developed higher antibody titers, as it has been reported that patients aged higher than 60 can develop this condition (11, 24). So higher antibodies in the elderly may simply reflect the likelihood of previous more severe SARS-CoV2 disease.

We also found that except for the Gam-COVID Vac, in the other vaccines requiring two doses, the first dose was sufficient to induce maximal antibody levels in subjects with a history of SARS-CoV-2, as previously shown for the BNT162b2 mRNA vaccine (9, 25–29)

For most patients, at least one AEFI occurred with all types of vaccines during the first 24 hrs. after injection, and most of them qualified the symptoms as “very mild” or “mild” both after the first and second dose, except for mRNA-1273 for which nearly half of the volunteers reported moderate symptoms after the second dose as reported previously (19, 20, 28, 30–34).

Our models showed a higher risk for women and young people to experience AEs. A real-world study that reported AEFI of patients receiving BNT162b2 mRNA, mRNA-1273, or Ad26COV2 in one or two doses (35), an independent studies conducted with Gam-COVID also confirmed this result (33, 36). The study of Ramasamy et al. on ChAdOx1 showed more adverse events in the group of 18-55 years old (20). The study conducted by Scott A et al. showed that severe adverse events (grade 5) were more frequent in women with Ad5-nCoV (32).

On one hand, for the Naïve SARS-CoV-2 cohort, the vaccines Gam-COVID-Vac, Coronavac, and ChAdOx1-S were related to fewer AEs after the second dose than BNT162b mRNA, and to the best of our knowledge, there are no studies that compare AEs across these types of vaccines. Also, the delta of antibodies was related to AEs after the second dose in Gam-COVID-Vac. As previously published, mRNA-1273 had higher AEFIs when compared to BNT162b2 mRN (37).

On the other hand, for subjects previously exposed to SARS-CoV-2, those receiving Gam-COVID-Vac showed a greater number of AEs than BNT162b mRNA, while subjects receiving Coronavac showed significantly fewer events when compared to BNT162b mRNA. In general, an increase in the antibody levels was related to an increase in the number of AEFIs, but for people receiving Gam-COVID-Vac or ChAdOx1-S, greater changes in antibody levels corresponded to fewer AEs. This last result should be taken with caution due to the limited number of patients falling in the category. To the best of our knowledge, this has not been previously shown. The study conducted by Sung Hee-Lim et al. that compared the antibody response with adverse events in patients vaccinated with BNT162b mRNA or ChAdOx1-S reported no association with antibody change and adverse events; however, patients previously infected with SARS-CoV-2 were excluded (38).

Interactions between age and vaccines were included for two main reasons: first, we decided to include in the model the vaccination strategies performed by the different countries that administered different vaccines according to age to correct for this possible confounding effect.

Second, our results show that with respect to BNT162b2 mRNA vaccine (where age showed a negative correlation with AEs), any other vaccines with a significant coefficient > 19.403 (e.g. Gam-COVID-Vac, ChAdOx1-S) will be correlated with AEs but with a positive correlation instead of a negative one like for BNT162b2 mRNA. Therefore, in comparison with BNT162B2 a higher level of antibodies in those who receive Gam-COVID-Vac, ChAdOx1-S or Coronavac are related to less AEs.

This study shows a low rate of infection in all vaccine groups. However, the highest rate of SARS-CoV-2 infection in this follow-up period was for the one-dose vaccines Ad5-nCoV and Ad26.COV2. Also, Coronavac had a high rate of infection. It is important to consider that the patients were followed for a short period.

As a limitation of our study, we recognize that we had a small sample size in some vaccine groups such as Ad5-nCoV; however, to the best of our knowledge, there is no other study where this type of vaccine has been compared to other types of vaccines in a real-world setting. Even though there was a standardization between countries in relation to AEFI definition and the method for registration of these events, there can be cultural differences that can affect the likelihood of the degree of reporting and registration of an AEFI event. Also, the personal sense of seeking for help and vaccination differs among participants and across health systems. In the future it will be of interest to follow the IgG titers for a longer period and to evaluate the effect of heterologous combinations of these vaccines.

In conclusion, this comparative study of vaccine types shows positive immunogenicity and seroconversion of BNT162b2 mRNA, mRNA-1273, Gam-COVID-Vac, Coronavac, ChAdOx1-S, Ad5-nCoV, and Ad26.COV2. The highest IgG response was for the mRNA vector vaccines and the lower for the inactivated vaccine. Women and young individuals developed more AEs. In the naïve SARS-CoV-2 cohort, the vaccines Gam-COVID-Vac, Coronavac, and ChAdOx1-S were related to fewer AEs after the second dose of BNT162b mRNA. For patients who received Gam-COVID-Vac, higher antibody levels after the second dose were related to a greater number of AEs. In people previously exposed to SARS-CoV-2, those receiving Gam-COVID-Vac showed a greater number of AEs than BNT162b mRNA, while subjects receiving Coronavac showed significantly fewer events when compared to BNT162b mRNA. In general, an increase in antibody levels was related to AEs.

## Data Availability Statement

The raw data supporting the conclusions of this article will be made available by the authors, upon reasonable request.

## Ethics Statement

This study was approved by the local Institutional Review Board of each institution (Universidad de Monterrey, Humanitas Clinical and Research Center, Fundação São Francisco Xavier, Ternium Health Center in Rio, Hospital Municipal San Jose, Hospital Interzonal de Agudos San Felipea) and conducted per the Code of Ethics of the World Medical Association (Declaration of Helsinki) for experiments that involve humans.The patients/participants provided their written informed consent to participate in this study.

## Author Contributions

Conceptualization: MR-I, MR, MT, MS-S, and EA. Formal analysis: MR-I, MR, MT, MS-S, AG-C, CP, RL, RS, MM, and RB. Investigation: MR-I, AG-C, MR, MT, MS-S, CP, RL, RS, RB, MM, DR-S, YH-R, AA-V, GD-P, IB-F, RG-F, EA, AA, MH-G, AB-B, CA, GP-B, MT, and MR. Resources: MR, MT, and MS-S. Writing – original draft: MR-I, AG-C, CP, MM, RS, RL, RB, MS-S, EA, DR-S, YH-R, AA-V, GD-P, IB-F, RG-F, EA, AA, MH-G, AB-B, CA, GP-B, MT, MR. Writing – review and editing: MR-I, AG-C, CP, MM, RS, RL, RB, MS-S, EA, DR-S, YH-R, AA-V, GD-P, IB-F, RG-F, EA, AA, MH-G, AB-B, CA, GP-B, MT, MR. Project administration: MR, MT, MS-S. Supervision: MR-I, MT, MR, and MS-S. Funding acquisition: MT, MR, and MS-S. All authors contributed to the article and approved the submitted version.

## Funding

This research was conducted using private funding from Techint Group of Companies. The funders had no role in study design, data collection, analysis, and decision to publish.

## Acknowledgments

We thank Erika Bienek (Techint Group Community Relations Director), Walter Bruno (Humanitas Communications Director), Cesar Bueno (Fundación São Francisco Xavier President), Mario Galli (Techint Group Communications Director), Pablo Sturiale (Apsot Director) for their support of the work and discussions.

## Conflict of Interest

The authors declare that the research was conducted in the absence of any commercial or financial relationships that could be construed as a potential conflict of interest.

## Publisher’s Note

All claims expressed in this article are solely those of the authors and do not necessarily represent those of their affiliated organizations, or those of the publisher, the editors and the reviewers. Any product that may be evaluated in this article, or claim that may be made by its manufacturer, is not guaranteed or endorsed by the publisher.

## Abbreviations

(AEFI), Adverse events following immunization; (AEs), Severe adverse events.
